# Rhythmic Music Training’s Effect on Syntactic Structure Processing among Malay Adolescents with Syntactic Specific Language Impairment

**DOI:** 10.21315/mjms2023.30.4.12

**Published:** 2023-08-24

**Authors:** Hui Ying Jong, Rozaida Abdul Rauf, Jafri Malin Abdullah, Faruque Reza, Jason Tye, Costas I Karageorghis, Garry Kuan, Peter Chong Jian Li, Norsofiah Abu Bakar

**Affiliations:** 1 School of Humanities, Universiti Sains Malaysia, Pulau Pinang, Malaysia; 2 School of Medical Sciences, Universiti Sains Malaysia, Kelantan, Malaysia; 3 Performance and Pedagogy, School of Arts, Universiti Sains Malaysia, Pulau Pinang, Malaysia; 4 Department of Life Sciences, Brunel University London, Middlesex, United Kingdom; 5 School of Health Sciences, Universiti Sains Malaysia, Kelantan, Malaysia; 6 School of Languages, Literacies and Translation, Universiti Sains Malaysia, Pulau Pinang, Malaysia

**Keywords:** adolescents, syntactic specific language impairment, rhythmic music training, which questions

## Abstract

**Background:**

Adolescents with syntactic specific language impairment (S-SLI) fail to comprehend *which* object questions. We hypothesised that rhythmic music training is more effective in treating this condition than conventional methods because music is often perceived as having a clear, isochronous beat or pulse. Thus, this study aims to investigate the effects of rhythmic music training on the syntactic structure processing of Malay *which* questions among native adolescents.

**Methods:**

In this research study, the participants were three groups of Malay adolescents aged 13 years old–15 years old: i) adolescents with S-SLI with music training, ii) adolescents with S-SLI without music training and iii) typically developing adolescents. Before and after music training, the participants were given a sentence-picture matching task. Accuracy measures and reaction times were captured using E-Prime 2.0.

**Results:**

The results indicated that with music training, the accuracy and reaction time associated with *which* object questions among the two SLI groups were significantly higher and lower, respectively.

**Conclusion:**

The implications of using rhythmic music training in enhancing syntactic structure processing are also discussed.

## Introduction

Specific language impairment (SLI) is a communication disorder that interferes with the development of language skills. SLI has also been described as a heterogeneous deficit that causes difficulties in various aspects of language, specifically word-finding, phonology, morphology, syntax, semantics and pragmatics (1–3). SLI is not currently diagnosable from a language user’s neurological etiology (2). Although children with SLI exhibit language impairments, their performance in nonverbal cognitive tasks remains within normative levels (2). Therefore, they are classified as children or adolescents with SLI, a classification that includes children who might differ considerably from one another in terms of their degree of language impairment.

The present study focused on syntactic SLI (S-SLI), which involves the production and comprehension of morphosyntax. Children with S-SLI have trouble processing sentences in which the object moves across the subject, resulting in a noncanonical order of arguments, as in the case of object relative clauses, object wh-questions and topicalised structures (4–7). In English, the canonical (i.e. simplest) word arrangement in sentences is subject-verb-object (SVO). Complex sentences in which noun phrases have been moved out of their canonical order, such as the passive voice (e.g. ‘The artist was chased by the thief’) and the object relative clause (e.g. ‘The artist that the thief chased’) (8), are rather difficult to comprehend (9). However, children with SLI perform above average with other syntactic structures, such as simple sentences (10) and sentences that maintain the canonical order of arguments (8).

The current standard treatment for S-SLI is based on the use of complex sentences known as the treatment of underlying forms (TUF) (9). Through complex sentence training, improvement was achieved not only in the targeted structures but also in other untrained structures. In addition to complex sentence training, musical training is another potential treatment for S-SLI. Several studies have suggested that musical abilities are associated with language competence (11–13). Forgeard et al.’s (14) study indicated that the effects of music training on language competence are causal in nature. Such effects have been observed widely among children with musical training (15–16). For example, many attempts have been made to demonstrate that musical ability is positively correlated with reading ability (17). Furthermore, rhythmic ability has been shown to be correlated with phonological and word-identification competence (18). Formal music training is associated with the enhancement of brain response to subtle acoustic variations in speech syllables and the superior acquisition of foreign language skills (19–20). In kindergarteners, it has been found that music training increases their phonological awareness skills more than sports training (21–24). Music training also tends to result in a better perception of linguistic pitch or better rhythmic sensitivity and reading skills (18, 24–28); it has also been shown to improve brain response to speech segmentation, syllabic duration, and voice onset time (25, 29–30).

Even though recent studies have indicated that SLI is associated with impairments in musical processing (31–33), several researchers have highlighted the potential utility of rhythmic musical training as part of SLI therapy to address speech and language difficulties (16, 25, 34). This recommendation of rhythmic musical training (35) was based on Patel’s scholarly work (36), which highlighted several similarities between music and language in hierarchical syntactic structures. Such similarities between language and music may influence the sharing of their processing resources. For instance, the broad notion of language ‘syntax’ can be investigated at several representational levels, and this syntax can also be adapted to musical forms. In a narrow sense, syntax concerns the set of rules and principles for combining words into larger units, such as phrases, clauses and sentences.

Musical syntax has both harmonic and rhythmic dimensions (37). Thus, the similarities between linguistic and musical syntactic processing can be attributed to an overlap in brain networks that provide shared resources for syntactic integration (38). Patel’s (36) notion of overlapping brain networks provides a premise for music training to influence language impairment, language acquisition, or even literacy. Cumming et al. (34) suggested that musical training, particularly in the SLI context, needs to be designed carefully to ensure that it is effective. The authors provided considerable insight into multimodal rhythmic music training for SLI, which can offer greater benefits than simpler beat-based training.

The overlap, precision, emotion, repetition and attention (OPERA) hypothesis (38) proposes that the benefits of musical training for the neural encoding of speech are driven by adaptive plasticity in speech-processing networks and that plasticity occurs when five assumptions are met: i) overlap—an anatomical overlap exists in the brain network during the processing of music and language; ii) precision—for precise processing, music places greater demands on these shared networks than speech; iii) emotion—musical activities that engage this network can elicit strong positive emotions; iv) repetition—musical activities that engage this network are repeated extensively; and v) attention—musical activities that engage this network are associated with focused attention.

In relation to the issue of whether music training should be conducted on an individual or group basis, many contemporary experts assert that it might be more meaningful to create an opportunity for social interaction through group-based music training (36, 38). Degé and Schwarzer (21) demonstrated the efficacy of music training in addressing both physical and psychosocial needs among hospitalised children. The authors concluded that music is a non-threatening and pervasive medium that provides a safe context for such children. The study also found that music training is of comparable significance to physical therapy, occupational therapy, speech and language therapy and recreational therapy.

Although the extant music therapy literature suggests that a strong association exists between language and music (18, 30), it is difficult to make definitive claims about the effects of rhythmic music training on syntactic structure processing. Empirical evidence that favours such a highly specific relationship between language and music is presently lacking. However, the integration process mentioned above provides a novel insight that underpins the present study, focusing on rhythmic music training and leading to a testable prediction that rhythmic music training may enhance the comprehension of syntactic structures in S-SLI.

## Methods

### Study Design

This study used a quantitative design. The data were collected only once.

### Participants

A total of 67 adolescents aged 13 years old–15 years old, comprising the S-SLI group (with and without music training) and the typically developing (TD) group, volunteered to participate in this study. All participants were students of the Malaysian public secondary school system. Due to the omission of SLI from the fifth edition of the Diagnostic and Statistical Manual of Mental Disorders (DSM-5), the identification of adolescents with S-SLI was based on the assessment of ‘deviation from normal’, as determined by the results from a battery of language tasks. An initial screening test determined that the participants met all the exclusionary criteria for SLI (34, 35): i) no hearing impairments and no recent episodes of otitis media; ii) no abnormalities in oral structure or problems in oral function; iii) no evidence of obvious neurological impairment or impaired neurological development and iv) no symptoms of impaired reciprocal social interaction or restriction of activities typical of autism or pervasive developmental disorders. SLI is a condition characterised by a child’s failure to develop spoken language according to the typical developmental schedule without any apparent physical cause. Possible physical causes include hearing impairment and recent episodes of otitis media (in the middle ear) or physical impairment of articulators (such as cleft lip and/or palate), which are considered to impact language development and may result in language delay or language disorders. If the child presents with any of these problems, it would preclude a diagnosis of SLI (2). Furthermore, SLI does not arise from obvious neurological impairment or impaired neurological development. This criterion was used to exclude language problems arising from an identifiable or diagnosed congenital or acquired disorder equivalent to a circumscribed brain injury. SLI-diagnosed children have age-appropriate skills in areas such as skills of daily living and nonverbal ability. Furthermore, autism was also excluded from an SLI diagnosis. The typical presentation of SLI involves a child with normal social interaction and nonverbal communication but facing specific difficulties in mastering the structural aspects of language, especially in syntax and phonological skills.

A second test ensured that participants’ nonverbal intellectual functioning was at an age-appropriate level, as indicated by scores on Raven’s (39) matrices test, in which they performed within 1 standard deviation (SD) of the average for adolescents their age. The use of nonverbal intelligence measures to define SLI is fundamentally tied to one’s theoretical conceptualisation of the relationship between language and other aspects of cognitive development. The third test administered was a clinical language assessment of the participants’ syntactic abilities. To this end, the participants were categorised into either the S-SLI or TD group based on their performance in four syntactic comprehension and production tests and one repetition test.

The required sample size was estimated using G*Power version 3.0.10. With a hypothesised effect size of 0.65, an alpha level of 0.05, a power of 0.80, three groups, and two measurement occasions, the estimated required minimum sample size was 27. Assuming an estimated dropout rate of 10%, a sample of 30 participants was deemed sufficient to detect the magnitude of the within-between differences expected. The final set of participants comprised 20 S-SLI adolescents (10 in a rhythmic music training group and 10 in a control group) and 10 TD participants. Neither the S-SLI control group nor the TD group underwent any training.

### Syntactic Screening Tests

**Figure 1:**
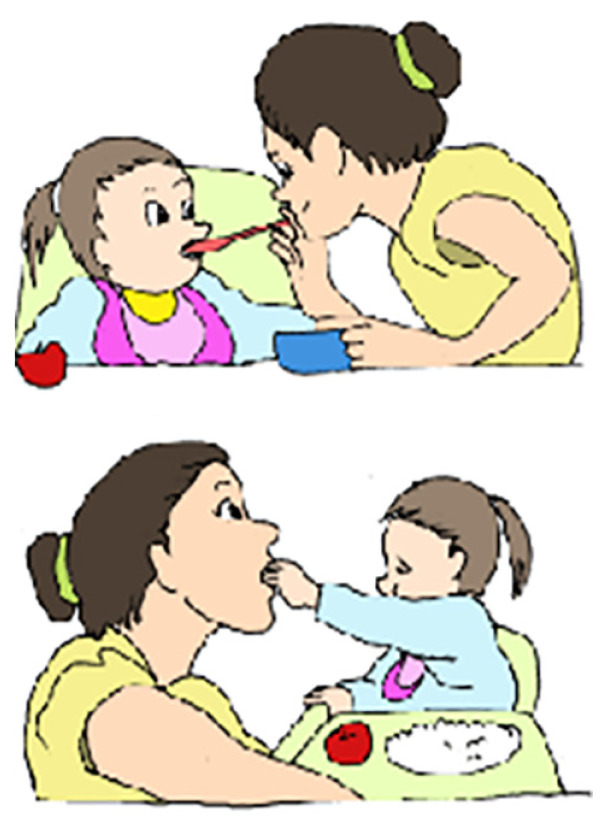
Example of a binary picture-matching task

The diagnostic criteria for SLI, on the whole, consisted of test scores. In morphosyntax, tasks focusing on embedding and/or syntactic movement are expected to assist researchers in identifying adolescents with S-SLI. In this study, the syntactic screening tests included syntactic structures that had previously been demonstrated to detect S-SLI (8). The comprehension of relative clauses was assessed using a binary sentence-picture matching task (BAMBI ZTI) and task-involving comprehension questions (BAMBI ZIKA MEGUVANA MEGUVANA). In the binary sentence–picture matching task (BAMBI ZTI), 40 items containing object relative clauses and SVO sentences were presented. A native Malay speaker read semantically reversible sentences to each participant, who was presented with two pictures on the same page. The participants were asked to point to the picture that correctly described the sentence by selecting from two pictures displayed vertically, one above the other. In one picture, the roles matched the sentence, while in the other picture, the roles were reversed. All verbs were agentive and transitive. To preclude the semantic cue, the figures in each picture were always the same gender and number (e.g. a mother and a daughter; Figure 1).

The production of relative clauses was assessed using two types of elicitation tasks: i) a preference task and ii) a picture description task. In the preference tasks, a preference question was used to evoke the production of a subject and object relative clause. Half of the preference tasks elicited a subject relative clause (six elicited subject relative clauses) and the other half elicited an object relative clause (six elicited object relative clauses). The order of the two types of relative clause target sentences was randomised. A supplementary task used to elicit both relative clauses was a description of picture pairs, similar to the picture in Figure 1, wherein one picture described one figure performing an action on the other. In the second picture, the roles were reversed. The experimenter used simple sentences to describe the two pictures, and the participant was then asked about one of the figures and its role in each of the pictures. The target response was either a subject or object relative clause. There were 10 subject relative clauses and 10 object relative clauses in the picture-pairs description task. The order of the relative clauses was randomised between the pictures.

To assess each participant’s ability to repeat the relative clauses and understand their deficit, it is necessary to correlate the relative clauses with the syntactic or embedding clauses or with sentence length. It is a repetition test of subordinated and coordinated structures. The test included 24 sentences of three types: i) eight object clauses, ii) eight coordinated sentences, and iii) eight sentential complements. All the participants heard a sentence and were asked to count to 10 aloud before repeating the sentence.

Failure in a test was defined as a performance that was significantly poorer than the mean score obtained by the TD group. Thus, the participants were tested using Crawford and Garthwaite’s (40) method and the receiver operating characteristic (ROC) curve to compare an individual subject to a group, with an alpha level of 0.05 in the test-test condition, in which the performance of the five diagnostic tests was evaluated using the measures of sensitivity and specificity. In the binary sentence-picture matching task (BAMBI ZTI), 55% of the participants failed to comprehend object relatives. However, only 27% and 18% of the participants failed to comprehend subject relative clauses and SVO sentences, respectively. In the second comprehension task (BAMBI MEGUVANA), the comprehension of subject relatives and SVO sentences was considerably better, with only 3% and 4% of the participants failing in these two types of sentences, respectively, compared with 37% of the participants who failed to comprehend object relatives.

In the first production test of the elicitation task (preference task), 25 of the 67 participants failed in the object relative clause condition, while 52 of 67 participants failed in the object relative clause condition in the second production test (picture description task). For the repetition test, repetition of coordinated sentences (29 of the 67 participants failed in this test) was significantly better (*P* < 0.05) than repetition of object relative clauses (29 of 67 participants failed this test) and sentential complements (29 of 67 participants failed in this test). Quantitatively, the participants made the same number of errors in the tasks involving object relatives and sentential complements. Finally, data from syntactic screening tests were compared with the diagnostic reports that the speech therapist provided. The speech therapist also agreed with the grouping of the three research conditions for this study. Based on the participant’s performance in the object relative clause conditions, 20 participants were grouped under the S-SLI category, while the remainder were assessed as TD speakers. The students’ teachers were consulted while grouping some of the students. From the S-SLI students, 10 were randomly selected into the musical training group. Another 10 were randomly selected into the non-intervention S-SLI control group and 10 TD students were also randomly categorised into the typical control group.

### Measures

#### Comprehension of Wh-Questions

Before and after the rhythmic music training session, the comprehension of ‘manakah’ (*which*) and ‘siapakah’ (*who*) questions in subject and object forms was tested among control groups and experimental group using a binary picture-matching task (Figure 1) presented using E-Prime 2.0. The participants were given a total of 80 questions in the Malay language, 20 of each type (20 ‘siapa’ (*who*) subject questions, 20 ‘manakah’ (*which*) subject questions, 20 ‘siapa’ (*who*) object questions and 20 ‘manakah’ (*which*) object questions). Please refer to the examples below along with the matching picture:

- *Who* subject question
*Siapakah yang sedang memeluk seorang budak perempuan?*
  Who that is hug a girl
  *Who* is hugging a girl?
- *Which* subject question
*Budak perempuan manakah yang sedang memeluk seorang ibu?*
  Girl which that is hug a mother
  *Which* girl is hugging a mother?
- *Who* object question
*Siapakah yang seorang ibu peluk?*
  Who that a mother hug
  *Who* is the mother hugging?
- *Which* object question
*Budak perempuan manakah yang seorang ibu sedang peluk?*
  Girl which that a mother is hug
  *Which* girl is the mother hugging?

**Figure 2:**
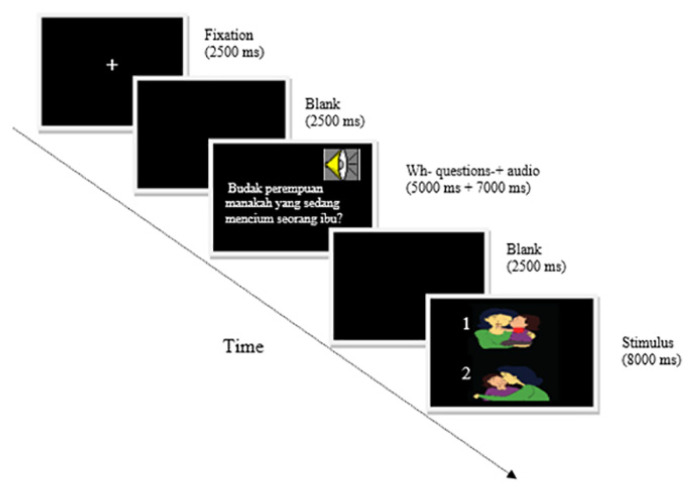
A block diagram of the algorithm showing the binary picture-matching task

Each participant was tested individually in a quiet room. The questions were posed in random order, and no more than two questions of the same type appeared consecutively. The participant saw each set of pictures four times, paired with each of the four question types; each question-picture set combination appeared once during each session. All verbs were in the agentive transitive form, and all questions were semantically reversible so that comprehension of the meaning of the words alone could not determine the sentence’s meaning (we did not use irreversible sentences, such as, “Which girl is eating a sweet?” – only reversible sentences, e.g. “Which girl is kissing her mother?”). For this task, the experimental questions followed a fixation symbol, ‘+’ (2,500 ms), a blank (2,500 ms) and a sequential presentation (5,000 ms) of one of the four question types. Following the auditory presentation of each question (7,000 ms), the picture set was displayed (8,000 ms) on the upper and lower sections of the laptop screen, and the participants were asked to press either ‘1’ (upper picture) or ‘2’ (lower picture) (Figure 2). The auditory presentations were recorded digitally using a native female Malay speaker. Measures of accuracy and reaction time (RT) were recorded.

### Music Selection

Eight songs were used as materials for musical training. The selection criteria were based on their familiarity to the general public and their inclusion of a prominent beat. All eight songs were used in all three training phases for every participant in the musical training group. The selected songs were ‘Twinkle, Twinkle, Little Star’, ‘See-Saw Up and Down’, ‘Hot Cross Bun’, ‘Good Night, Sleep Tight’, ‘Rasa Sayang’, ‘Snail-Sail’, ‘Apple Tree’ and ‘Where Is My Friend?’

### Procedure

The rhythmic music training was adapted from the auditory-motor mapping training (AMMT) method. This training consisted of 24 sessions of 40 min–45 min each, administered over an 8-week period. In the present study, AMMT training was divided into four phases. Several activities were used throughout these four phases, wherein the students could experience the musical beat through visual, oral and kinesthetics channels. Phase 1 aimed to teach the participants to move their bodies in conjunction with the beat. This included physical actions, such as clapping, nodding and tapping their feet. Simple rhythmic percussion activities were also assigned to help the participants feel the beat.

Phase 2 focused on training the participants to identify the pitch of the music with the aid of the beat. Phase 2 comprised three types of activities. The first activity consisted of singing to ensure that the participants could sing in tune and with the correct intonation. The second activity focused on beat accuracy while singing, while the third activity introduced rhythm identification. The participants were encouraged to play with rhythm instruments while singing during this activity.

In phase 3, the participants were trained to identify the rhythm without the aid of the beat. Using a mentor-mentee approach, the participants who were more proficient at rhythm identification in the third activity of phase 2 were designated as mentors. They were asked to maintain a steady rhythmic pattern by clapping, stomping or using easy-to-play percussion instruments (such as egg shakers or rhythm sticks). The rest of the participants were then designated as mentees. They were asked to pay attention to the rhythmic pattern that the mentor demonstrated. These mentor–mentee interactions were conducted one-on-one. Once a steady rhythm was established, the mentee shook the egg shaker to match the demonstrated rhythmic pattern. In a follow-up activity, the music therapist asked the participants to perform one set of movements, specifically clapping (while saying aloud ‘clap-clap-clap-clap’), patting both thighs with the hands (while saying aloud ‘tap-tap-tap-tap’), sliding one hand from arm to finger (while saying aloud ‘slide-slide-slide-slide’) and moving both palms upward four times (while saying aloud ‘go-go-go-go’). The clapping and patting of the thighs were repeated three times before the actions of sliding and moving both palms upward. In these rhythmic exercises, the participants practiced various beats, including quaver (clapping), crotchet (patting thigh), minim (sliding one hand from arm to finger) and semibreve (moving palms upward).

Phase 4 trained the participants to integrate all the musical skills learned in the previous phases. In a musical chair-style improvisation game, the participants were split into two groups: one group set the rhythm and the other group was supposed to join in the song without interrupting the established rhythm. Whoever made a mistake would be eliminated until there was finally one participant remaining from each group. When Group 1 clapped their hands for the second time, Group 2 started the exercise. During phase 4, the participants were given the chance to play musical instruments, including an egg shaker, maracas, a rattle, castanets or a triangle. These percussion instruments provide an ideal introduction to learning how to play a beat or rhythm (including pattern recognition). Musical instruments can also help cultivate social skills, build self-esteem and promote team building in the creation of music.

### Data Analysis

**Figure 3:**
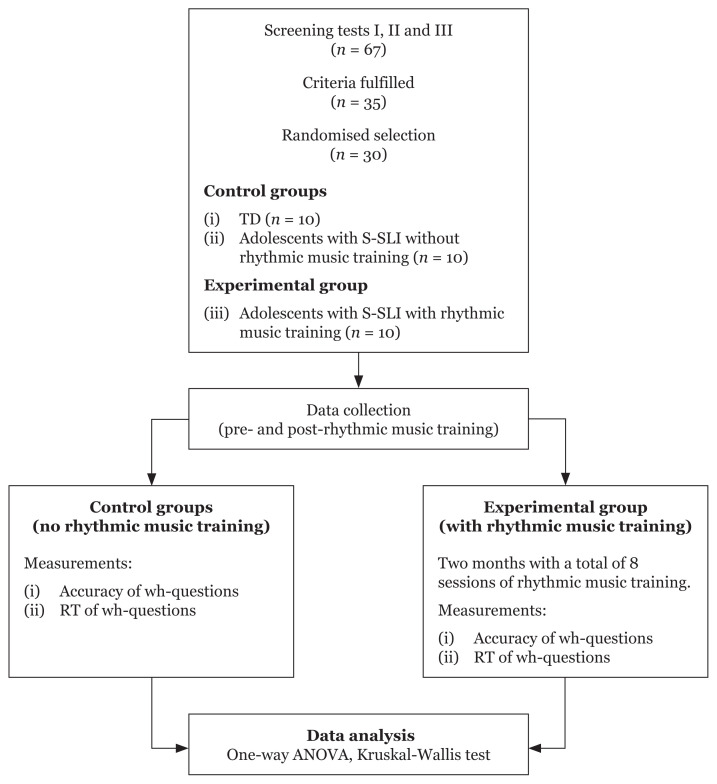
Flowchart depicting the study design

The Statistical Package for the Social Sciences (SPSS) version 26.0 was used to analyse the data and normality tests were conducted for each cell of each analysis. One-way ANOVA and pairwise comparisons were conducted on the gain score (post-test minus pre-test) of the RT for four types of wh-questions, while Kruskal-Wallis testing and pairwise comparisons were conducted on the gain score of accuracy and no response for the same four types of wh-questions. The Kruskal-Wallis test serves as the non-parametric alternative to the one-way, between-subjects analysis of variance. A flow chart of the study design is provided in Figure 3.

## Results

### Accuracy

The Kolmogorov-Smirnov normality test indicates a significant departure from the normal distribution for accuracy performance (*P* < 0.05). As a result, the non-parametric Kruskal-Wallis test was used to determine whether a significant difference existed across the groups in terms of performance accuracy. This test revealed a significant difference in accuracy for *which* object questions (*H*(30) = 16.53; *P* = 0.001) across the three groups of adolescents. However, there was no difference in the gain score for accuracy for the *who* subject questions (*H*(30) = 1.28; *P* = 0.530), *who* object questions (*H*(30) = 5.17; *P* = 0.080) and *which* subject questions (*H*(30) = 1.40; *P* = 0.500). Post-hoc Mann-Whitney U tests used to compare each combination of groups indicated that in *which* object questions, Group 3 was significantly different compared with Group 1 (*P* = 0.001; *r* = 3.45) and Group 2 (*P* = 0.010; *r* = −2.26).

### Response Time

A one-way ANOVA was used to examine differences across the three groups concerning the RT for answering wh-questions, as measured through the binary picture-matching task. There was a significant (*P* < 0.05) difference in RT for the four types of wh-questions in the three research conditions: i) *who* subject questions (*F*(2, 27) = 6.22, *P* = 0.01); ii) *which* subject questions (*F*(2, 27) = 4.73, *P* = 0.020); iii) *who* object questions (*F*(2, 27) = 8.41, *P* = 0.001); and iv) *which* object questions (*F*(2, 27) = 25.05, *P* = 0.001). Partial eta-squared effect size calculations yielded the following values: 0.32 for *who* subject questions, 0.26 for *which* subject questions, 0.38 for *who* object questions and 0.65 for *which* object questions.

Accordingly, the difference in the mean gain score RT of the four types of wh-questions among the groups was considerably large. Post-hoc comparisons were conducted using Tukey’s HSD test. For the *who* subject questions, the RT for Group 2 (*M* = −382.17, SD = 224.87) was significantly lower (*P* = 0.001) than that for Group 1 (*M* = 4.70, SD = 292.35) and Group 3 (*M* = −91.69, SD = 244.09). For the *which* subject questions, the RT of Group 1 (*M* = 43.35, SD = 305.71) was significantly (*P* = 0.0001) different from that of Group 2 (*M* = −292.32, SD = 184.47). For the *who* object questions, the RT of Group 2 (*M* = −572.92, SD = 290.64) was significantly (*P* = 0.001) different from that of Group 1 (*M* = −130.05, SD = 220.91) and Group 3 (*M* = −119.01, SD = 325.43). For the *which* object questions, Groups 1 (*M* = −8.60, SD = 305.62) and 2 (*M* = −132.11, SD = 498.19) had significantly (*P* = 0.001) different RTs than that of Group 3.

### No Response

The Kolmogorov-Smirnov normality test also indicated a significant non-normal distribution for ‘no response’ (the absence of a response). As a result, the Kruskal-Wallis test was used to examine differences across groups in the no-response category. This test revealed a statistically significant difference in the no-response category for *which* object questions (*H*(30) = 15.00, *P* = 0.001) and *who* subject questions (*H*(30) = 6.04, *P* = 0.050) across the three groups. The test also indicated no significant difference in the gain score of accuracy relating to *who* object questions (*H*(30) = 2.46, *P* = 0.290) and *which* subject questions (*H*(30) = 2.00, *P* = 0.370). For the *which* object questions, pairwise comparisons indicated that Group 3 was significantly different from Group 1 (*P* = 0.001, *r* = 3.13) and Group 2 (*P* = 0.010, *r* = 2.24). For the *who* subject questions, pairwise comparisons indicated that Group 2 was significantly different from Group 1 (*P* = 0.030, *r* = 1.32) and Group 3 (*P* = 0.04, *r* = −1.30).

## Discussion

The present study investigated the potential influence of rhythmic music training on the syntactic processing of wh-questions among adolescents with S-SLI. The SLI participants were tested using a language-based task and demonstrated weaker performance in wh-questions (accuracy, RT or no response) when compared with the TD group. Nonetheless, a notable finding is the difference between S-SLI adolescents with and without music training: superior performance was identified in the comprehension of wh-questions following rhythmic music training.

This result concurs with previous studies that reported an association between the auditory perception of sequential information (linguistic or non-linguistic) and language processing (41). The present findings also help support the notion that developmental language disorders are associated with a more general procedural deficit (18, 29, 42). The procedural component in the declarative/procedural model (42) is concerned with the learning and processing of context-dependent stimulus-response rule-like relations, mainly in temporal sequences (e.g. syntax, morphology, phonology and music). Our results are also in line with those of Gordon et al. (43), who suggested that the relationship between rhythm perception skills and grammar production skills was robust in 6-year-old participants. Kotz et al. (41) elucidated the beneficial effects of rhythm on syntactic processing among patients with basal ganglia lesions or Parkinson’s disease. Such patients bear similarities to children with SLI (and those with dyslexia) due to their impairment in temporal processing (34). Even though such processing is generally impaired, it is not entirely inhibited. Przybylski et al. (35) emphasised how decreased functionality is more likely to affect sequencing and segmentation (syntax) aspects of language processing because rhythmic and metrical structures are implemented less in language than in music. However, it is worth noting that musical stimuli can activate the impaired system, as music has a clear metrical structure that provides predictable cues and serves to stimulate internal oscillators, with attendant benefits for syntactical processing.

Given that linguistic difficulties in SLI are associated with prosodic structure, the benefits derived from the suprasegmental sequences of music were used to improve syntactic structures. Cumming et al. (34) stressed that individuals with pure SLI have a grammatical, but not phonological, deficit; therefore, motor synchronisation (beat) was unable to elicit benefits in grammatical development. Multimodal rhythmic music training (including visual modality) offers a benefit that transcends unimodal beat-based training (44). Therefore, Cumming et al. (34) suggested that the use of multimodal rhythmic training, with an emphasis on visual prosody (the mouth, jaw, cheek, and head movements that the speaker unconsciously produces when emphasising oral prosody), can be crucial to grammatical development. Oral prosody concerns variations in loudness duration, pitch, and speech pauses (33). Thus, if visual prosody is crucially important for grammatical development, Cumming et al. (34) implied that musical training involving group singing or other musical activities can offer circumstances for visual, auditory, and motor rhythmic synchronisation. This will lead to constructive outcomes in syntax recovery among children with SLI.

In the present study, a form of rhythmic music training known as AMMT was used to remediate syntactic processing impairment among adolescents. For the effectiveness of multimodal AMMT to be optimal, the training should engage participants in activities that combine physical, social, sensory, and cognitively challenging activities, as this combination can greatly improve conditions for neuroplastic changes in the rehabilitation recovery process (19). Considerable overlap exists between the present results and those of previous studies, which also revealed relationships between music processing and language (35). Upon closer inspection, it was found that the application of musical (rhythmic) sequences in this study matched the overall syntax structure of wh-questions.

**Figure 4:**
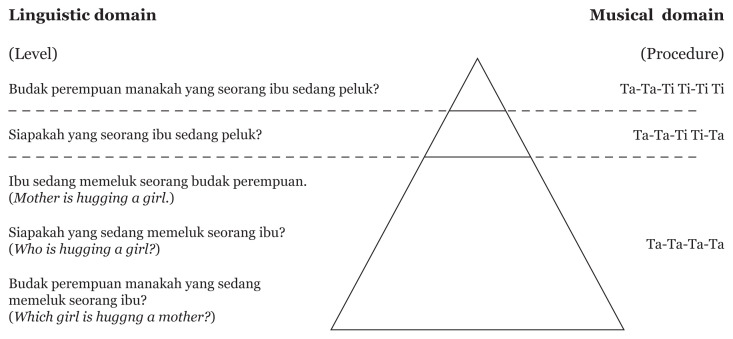
Three levels of the linguistic domain and three procedures for the musical domain

In Figure 4, the role of rhythmic music training in addressing language deficits in adolescents with S-SLI is visualised, particularly in terms of the similarity between the structure of the language domain and the musical domain. There are three distinct levels of linguistic structure that correspond to three distinct musical procedures. In music, when learning rhythm using the Kodály method, simple syllables will be used to represent the key rhythm (45); for example, one sound on a beat is called ‘Ta’, and two sounds on a beat are called ‘Ti Ti’ (44–45). The first procedure in the pyramid shows the basic or fundamental unit of music structure—namely, the crotchet beat (quarter note), which was easily picked up and played by the TD group and even the syntactic SLI group. Similar rhythmic characteristics can be found in language, as in the case of simple sentences (SVO) and wh-subject questions (please see the examples below).

(a) Ta-Ta-Ta-Ta
(b)
  - Ibu sedang memeluk seorang budak perempuan.
    Mother is hug a girl
    Mother is hugging a girl.
  - *Siapakah sedang memeluk seorang budak perempuan*?
    Who is hug a girl
    Who is hugging a girl?
  - *Budak perempuan manakah yang sedang memeluk seorang ibu?*
    Which girl that is hug a mother
    Which girl is hugging her mother?

The structure of the first level of language and music and the first method reflected a fundamental structure. As a result, the first level was less complex relative to the structure, which was apparent in the next two levels/procedures. For example, the same element of ‘Ta’ appeared from the beginning to the end of the rhythmic structure: ‘Ta-Ta-Ta-Ta’. This condition was similar to the canonical structure in a simple sentence and a wh-subject question. In the second musical procedure, the TD group and S-SLI adolescents were still able to tap the rhythm correctly, even though the rhythmic pattern was more difficult than in the first procedure (e.g. ‘Ta-Ta-Ti Ti-Ta’). The presence of the third element, ‘Ti Ti’, was somehow derived from ‘Ta-Ta-Ta-Ta’ in the first procedure and demonstrated a different surface rhythmic structure. The rhythmic structure in the second procedure is relatively similar to the wh-object *who* question, mainly in the movement or transition from one element to other elements, as illustrated in the following examples:

(c) Ta-Ta-Ti Ti-Ta
(d) [Siapakah]_i_ yang *seorang ibu* peluk t?
  Who that a mother hug
  Who is the mother hugging?

In example (c), the presence of the element ‘Ti Ti’ between the two occurrences of ‘Ta’ required the transition of ‘Ta’ from the second position to the fourth position, which moved across the ‘Ti Ti’ element. In example (d), the presence of the element ‘seorang ibu’ between the moved phrase ‘siapakah’ and trace ‘t’ required the assignment of a thematic role from ‘siapakah’ to the trace ‘t’. This type of thematic role assignment needed to extend across the ‘seorang ibu’. In language, when two arguments differ, such as when ‘siapakah’ is referential and ‘seorang ibu’ is nonreferential, one does not block the other. In rhythmic music training, the S-SLI adolescents were introduced to several techniques to enable them to recognise the ‘Ti Ti’ sound. The rhythm recognition activities were meaningful to them, as they helped them tap the ‘Ti Ti’ in ‘Ta-Ta-Ti Ti-Ta’ at the correct tempo. The reinforcement of learning rhythm in the second procedure also involved the position change of the element ‘Ti Ti’. By way of illustration, the ‘Ti Ti’ element was moved from the second position to the first (‘Ti Ti-Ta-Ta-Ta’), third (‘Ta-Ta-Ti Ti-Ta’), or fourth position (‘Ta-Ta-Ta-Ti-Ti’). When S-SLI adolescents mastered the skill sufficiently, they were allowed to advance to the next procedure, which comprised two elements of ‘Ti Ti’ in the rhythmic structure—for example, the appearance of ‘Ti Ti’ in the second and third positions (‘Ta-Ti Ti-Ti Ti-Ta’). The rhythmic structure in the third procedure is relatively similar to wh-object *which* questions, as illustrated in the examples below.

(e) a-*Ti Ti*-*Ti Ti*-Ta
(f) [Budak perempuan manakah]_i_ yang *seorang ibu* sedang peluk t?
  Which girl that a mother is hug
*Which girl is the mother hugging?*

In the example (e), the presence of two ‘Ti Ti’ elements between two occurrences of ‘Ta’ required the transition of ‘Ta’ from the second position to the fourth, which moved across two elements of ‘Ti Ti’ simultaneously. The ‘seorang ibu’ element’s presence in example (f) between the moved ‘Budak perempuan manakah’ phrase and trace ‘t’ required the assignment of a thematic role from ‘siapakah’ to the trace ‘t’ needed to across the ‘seorang ibu’ element. When two arguments are similar, such as the moved phrase ‘budak perempuan manakah’ and the intervening ‘seorang ibu’ element, both being referential, the assignment of the thematic role to the moved ‘budak perempuan’ phrase is prevented. This justifies why *who* questions do not pose problems for adolescents with S-SLI, but *which* questions do.

## Conclusion

We envisaged that the present study could add to the music and language literature regarding the use of musical training for adolescents with S-SLI. The findings demonstrated that such training significantly enhanced accuracy and shortened RTs in comprehension tasks involving wh-questions. Music training also reduced no-response answers to wh-questions. Furthermore, it provides novel insights into how music-related tasks, as opposed to speech-related tasks, can be integral to language interventions for adolescents with S-SLI. We recommend that further research examine different SLI forms. Moreover, more SLI studies from multi-cultural perspectives could confirm the benefits of using rhythmic music training to facilitate the syntactic processing of wh-questions.

## Limitations

The principal limitation of this experimental approach is the use of non-standardised, adapted, and translated imported screening tests due to the lack of formal and standardised screening tests in Malaysia (46–48). However, these non-standardised screening tests are some of the most widely used for identifying children or adolescents with SLI. The current study was also limited by the small number of participants. Nonetheless, it enabled us to implement a music intervention and understand the effectiveness of rhythmic music training on Malay adolescents.
